# On-the-road driving performance after use of the antihistamines mequitazine and l-mequitazine, alone and with alcohol

**DOI:** 10.1007/s00213-016-4386-7

**Published:** 2016-08-04

**Authors:** N. N. J. J. M. van der Sluiszen, A. Vermeeren, S. Jongen, E. L. Theunissen, A. C. M. van Oers, C. J. Van Leeuwen, A. Maret, C. Desforges, A. Delarue, J. G. Ramaekers

**Affiliations:** 1 Department of Neuropsychology and Psychopharmacology, Faculty of Psychology and Neuroscience, Maastricht University, P.O. Box 616, Maastricht, 6200 MD The Netherlands; 2 Institut de Recherche Pierre Fabre, Ramonville, France

**Keywords:** Antihistamine, Alcohol, L-mequitazine, Mequitazine, Highway driving test

## Abstract

**Objective:**

Previous studies demonstrated that mequitazine produces mild sedation after single doses.

Its enantiomer, l-mequitazine, has a stronger potency for the H1 receptor. The aim of the current study was to assess the effects of l-mequitazine and mequitazine, alone and with alcohol, on driving.

**Methods:**

Twenty-five healthy volunteers were treated with l-mequitazine 2.5, 5.0 and 10 mg, mequitazine 10 mg and placebo, alone and in combination with alcohol in a double-blind crossover design. Driving performance was assessed using the standardized highway driving test in normal traffic. Its primary measure is the Standard Deviation of the Lateral Position (SDLP). Secondary measures consisted of an auditory word learning test during driving, and subjective measures of driving performance.

**Results:**

L-mequitazine 2.5 and 5.0 mg showed no effect on SDLP in the highway driving test, while SDLP significantly increased after l-mequitazine 10 mg (alone +1.59 cm; with alcohol +1.41 cm) and mequitazine 10 mg (with alcohol +1.17 cm). Alcohol significantly impaired all performance measures (SDLP +2.63 cm) but did not interact with the effects of treatment. Subjective measures indicated that participants were aware of the impairing effects of alcohol, but not of l-mequitazine and mequitazine.

**Conclusion:**

L-mequitazine can be considered safe to drive in dosages of 2.5 and 5.0 mg. L-mequitazine 10 mg led to mild driving impairment. Alcohol impaired all performance measures and added to the effects of l-mequitazine and mequitazine.

## Introduction

In the general population, the prevalence of allergic rhinitis (AR), one of the most common chronic diseases, is 20–30 % (Nathan et al., 1997; Steerenberg et al., 2000). Untreated allergic conditions do not only affect a patient’s health, but also have a negative effect on cognitive functioning (Vuurman et al., 1993), quality of life (Kremer et al., 2002; Meltzer, 2001; Wallace et al., 2008) and driving performance (Vuurman et al., 2014). Antihistamines are the most commonly used pharmacotherapeutic option for treatment of AR. These medicinal drugs cause a relief of symptoms by antagonizing peripheral histamine 1 (H_1_) receptors. However, antihistamines can also cross the blood-brain barrier and block H_1_ receptors in the brain (Simons et al., 2011). Central H_1_ receptors are implicated in the maintenance of wakefulness (España et al., 2011). Consequently, H_1_ antagonists can also produce sedation, fatigue and associated performance impairment, which affects daytime functioning such as driving a car. This in turn leads to an increased risk for occupational injuries and traffic accidents (Gilmore et al., 1996; Nolen, 1997; O’Hanlon et al., 1995).

Sedation is of primary concern when considering the adverse effects of antihistamines. Older, first-generation antihistamines easily penetrate the blood-brain barrier and have shown to significantly impair performance in psychomotor and driving tests (McDonald et al., 2008). Newer, second-generation antihistamines penetrate the brain to a lower extent, due to their physico-chemical properties (Passalacqua et al., 1996; Tashiro et al., 2002). Therefore, these produce minor or no impairment on tests measuring memory, attention, psychomotor performance (Kay and Harris, 1999) and on-the-road driving, when given in the recommended dose (Isomura et al., 2014; Theunissen et al., 2009).

Mequitazine is a second-generation antihistamine with relatively high affinity for muscarinic receptors (Devillier et al., 2015; Kubo et al., 1987). Its effects on driving performance have been assessed in two studies with healthy volunteers (Theunissen et al., 2006; Theunissen et al., 2004). Dose-dependent driving impairment was found after mequitazine 5, 10 and 15 mg (Theunissen et al., 2004). A follow-up study with repeated doses of mequitazine 10 mg demonstrated mild driving impairment after the first dose, which disappeared within 8 days of dosing (Theunissen et al., 2006). It was concluded that mequitazine leads to mild impairing effects, compared to other second-generation antihistamines.

Mequitazine is a racemic mixture that comprises of two enantiomers, l-mequitazine (V0114) and d-mequitazine (V0162) (Devillier et al., 2015; Gauthier et al., 2014; Latil et al., 2007). The antihistaminergic activity mainly resides in the S-enantiomer, l-mequitazine, whereas the anticholinergic activity mainly resides in the D-enantiomer. In vitro binding studies have shown that the affinity of l-mequitazine for H_1_ receptors is approximately ten times higher and to muscarinic receptors ten times lower, compared to d-mequitazine (Heusler et al., 2015; Neliat, 2004). This binding profile could result in an increase of antihistamine activity and a decrease of anticholinergic side effects for l-mequitazine as compared to mequitazine.

Sedating antihistamines may potentiate the impairing effects of alcohol (Barbanoj et al., 2006; Hindmarch and Bhatti, 1987). Therefore, regulatory warnings concerning the combined use of these medicinal drugs and alcohol on operation of automobiles and other potentially dangerous machinery are applied to all first-generation antihistamines. Second-generation antihistamines, however, have not been found to potentiate the effects of alcohol (Vermeeren and O’Hanlon, 1998; Vermeeren et al., 2002a). Therefore, regulatory warnings concerning use of alcohol and driving are waived for these drugs (McDonald et al., 2008).

The aim of the present study was to assess the effects of single doses of 2.5, 5.0 and 10 mg l-mequitazine, alone and with alcohol, on highway driving and cognition. Effects of l-mequitazine and mequitazine 10 mg were compared to those of placebo. Performance was assessed using a standardized highway driving test and an auditory word learning test during driving.

## Methods

### Participants

Healthy male and female volunteers (age range 21–45 years) were recruited via poster advertisement and advertisements in local newspapers.

Participants were required to be in good health as confirmed by a medical history questionnaire, physical examination, a 12-lead electrocardiogram (ECG), blood chemistry and haematology and urinalysis. In addition, participants could only enrol when they possessed a valid driving licence for more than 3 years, had an average driving experience of at least 5000 km per year and a body mass index within the range of 19 to 30 kg/m^2^.

Volunteers were excluded for any of the following: pregnancy or lactation; history of severe physical or mental disorders, alcoholism or drug abuse; use of systemic medication within the previous month, except oral contraceptives; blood donation or participation in any other clinical trial within the previous 3 months; excessive caffeine use (>six standard units a day), mild smoking (>four cigarettes a day) or overconsumption of alcohol (>21 standard units a week) and total alcohol abstinence. In addition, cytochrome P450 CYP 2D6 slow metabolizers, as determined by a dextromethorphan test, were excluded from the study to avoid carry-over effects due to residual plasma concentrations or a dramatic increase of the washout periods.

Participants agreed not to use drugs of abuse or systemic medication (except oral contraceptives, aspirin and acetaminophen) from 2 weeks before treatment days until their completion. They had to refrain from smoking and/or consuming alcohol from the time of arrival at the site during treatment days until the completion of the testing day. In addition, alcoholic drinks, grapefruit juice and grapefruit were not permitted from 24 h before arrival. Caffeine was limited to one cup of tea at breakfast on treatment days.

The study was approved by the Ethics Review Committee of Maastricht University and the Academic Hospital Maastricht, and was conducted in accordance with Good Clinical Practices (CPMP/ICH/135/95) and with the code of ethics on human experimentation established by the Declaration of Helsinki (1964) and subsequent amendments. Written informed consent was obtained from each participant before enrolment.

### Study design and drug/alcohol administration

The study followed a randomized, double-blind, placebo controlled, five-period crossover design. Treatments were single oral doses of l-mequitazine 2.5, 5.0 and 10 mg, mequitazine 10 mg and placebo, alone and with alcohol. Medication was supplied in identical-appearing gelatine capsules. Treatments were administrated on five testing days, separated by washout periods of at least 7 days. On each treatment day, participants performed two driving tests. The first driving test was completed after drug or placebo treatment alone, and the second driving test was completed after an additional alcohol challenge. Alcohol administration always occurred after completion of the first driving test. Drug and placebo orders were randomly assigned from those residing in five, 5 × 5 Williams Squares.

The alcohol dosing regimen was developed to achieve and sustain a blood alcohol concentration (BAC) just under the local legal limit for drivers (i.e. 0.5 mg/ml) at the scheduled start of the second driving test. This normally required the administration of three weight and gender-calibrated doses of alcohol (0.23, 0.14, 0.14 mg/kg for males and 0.21, 0.13, 0.13 mg/kg for females) at 15-min intervals. Ethyl alcohol (96 %) was mixed with orange juice. The individual BAC was estimated from concentrations in expired alveolar using a Lion Alcoholmeter® SD-400 (Lion Laboratories Ltd., UK). Participants failing to achieve a BAC of 0.45 mg/ml after 1 h were given an additional dose of alcohol (0.05 mg/kg).

### Assessments

#### Highway driving test

In the standardized highway driving test (O’Hanlon, 1984), participants drive a specially instrumented car over a 100-km (61 miles) primary highway circuit accompanied by a licenced driving instructor having access to dual controls. The participant’s task is to maintain a constant speed of 95 km/h (58 mph) and a steady lateral position between the delineated boundaries of the slower right traffic lane. The vehicle’s speed and lateral position relative to the left lane delineation is continuously recorded. These signals are digitally sampled at 4 Hz and edited offline to remove data recorded during overtaking manoeuvres or disturbances caused by roadway or traffic situations. The remaining data yields the standard deviation of lateral position and speed for each successive 5-km segment and, as the square root of pooled variance over all segments, for the test as a whole. The primary outcome variable is the Standard Deviation of Lateral Position (SDLP, in cm) which is a measure of road tracking error, or ‘weaving’. The clinical relevance of performance changes in the highway driving test have previously been determined by establishing the relationship between blood alcohol concentration (BAC) and SDLP (Louwerens et al., 1987). An average increase in SDLP, compared to placebo, of +2.4 cm is comparable to driving under the influence of a BAC of 0.5 mg/ml and from that point onwards associated with a significant higher risk of traffic accidents (Borkenstein et al., 1974). The secondary outcome variable is the Standard Deviation of Speed (SDSP), which is an index of the ability to maintain a constant speed. The highway driving test has been proven sensitive to many sedating drugs and alcohol in blood concentrations as low as 0.35 mg/ml (Vermeeren et al., 2002a; Vuurman et al., 1996).

#### Auditory word learning test

During the driving test, participants performed an Auditory Word Learning Test (AWLT, adapted from (Rey, 1964)) to assess the effects on declarative episodic memory. In this test, participants were presented a series of 15 monosyllabic nouns through a loudspeaker in the car at a rate of one per 2 s. Immediately thereafter, they were required to verbally recall as many words as possible. The same series was repeated on two more trials, and the words recalled were recorded for offline scoring using a voice recorder. The sum of the number of words correctly recalled on the three trials was the Immediate Recall score. After a 20-min delay, participants were required to recall again as many words as possible without prompting. The number correctly recalled was the Delayed Recall score. Segment of the highway driving test, in which participants performed the AWLT, were removed during offline editing.

#### Subjective evaluations

Subjective evaluations of alertness and driving quality were assessed using a series of visual analogue scales (100 mm). Participants rated their expected driving impairment and completed a 16-item mood scale from which one factor was derived: alertness (Bond et al., 1974). Immediately upon conclusion of both driving tests, participants rated the quality of their driving performance.

### Procedure

**Fig. 1:**
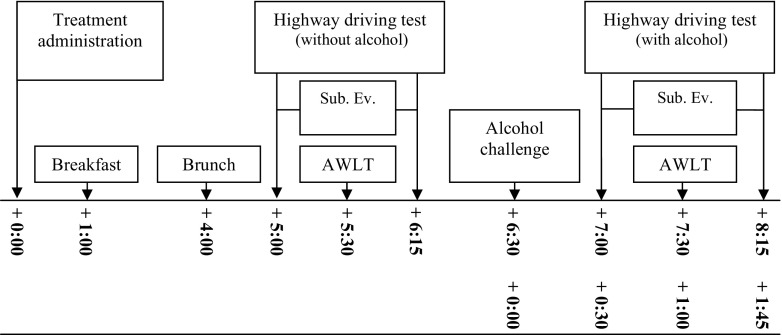
Overview testing day. Time (in hours) is displayed for post-treatment (*upper row*) and post- alcohol (*lower row*) administration. *Sub.Ev.* subjective evaluations, *AWLT* auditory word learning task

All participants were individually familiarized with the tests and procedures and rehearsed the driving test within a week before the first treatment day. On treatment days, participants were interviewed to verify eligibility and occurrence of adverse events since the last visit. They ingested their medication in the presence of an investigator at 8 am. They were required to fast from 3 h before until 1 h after ingestion to keep absorption rate and *t*_max_ before the driving tests constant. At 9 am, a standardized breakfast was served, and a light brunch at 12 am. All subjects conducted two successive highway driving tests on each testing day. The first test started at 1 pm (i.e. 5 h after drug or placebo treatment), and the second test started at 3 pm, (i.e. 7 h after drug or placebo administration and 0.5 h after additional alcohol consumption). Upon completion of the second driving test, participants were transported home. An overview of a testing day is given in Figure 1.

### Statistical analysis

A power calculation for repeated measures revealed that a sample of 25 participants resulted in a power of 99.9 % to detect a clinically relevant difference of 2.4 cm in SDLP between drug and placebo conditions, assuming an alpha of 0.05, a test-retest reliability for SDLP of 0.8 (Verster et al., 2011), an effect size of 0.81 and a within-subjects SD of 2.97 cm (Theunissen et al., 2013).

All measures were analyzed using general linear model (GLM) repeated measures. The model included two factors: Treatment (5 levels) and Alcohol (2 levels). Main effects of Treatment or Alcohol were further analysed by four drug-placebo contrasts. These simple contrasts were corrected with the step-down Holm-Bonferroni method (Holm, 1979). In addition, interaction effects of Treatment × Alcohol were assessed by using GLM repeated measures with both factors included. For each performance parameter, only participants with complete data sets were entered in the analysis. All statistical analyses were conducted by using the Statistical Package for the Social Sciences for Windows (version 21.0.01., SPSS Inc., Chicago, IL, USA).

## Results

A total of 31 volunteers were enrolled for this study. One withdrew after the first treatment period for reasons unrelated to the study, and five had to be replaced due to protocol violations of treatment orders. Twenty-five participants (12 males, 13 females) completed the study as planned. Their mean age was 33.4 ± 8.9 years. The men’s height and weight were 184 ± 7 cm and 78 ± 8 kg, and the women’s, 166 ± 6 cm and 67 ± 8 kg. Participants drove on average 12.756 ± 7909 km/year.

### Missing data and terminated driving tests

In total, eight out of 250 driving tests were not completed (3.2 %) because the driving instructor or the participant indicated that it was unsafe to continue. The driving instructor terminated five tests, one test after use of l-mequitazine 10 mg alone and four tests after use of alcohol (all of female participants), of which one in combination with l-mequitazine 2.5 mg, one in combination with l-mequitazine 5 mg and two in combination with l-mequitazine 10 mg. One participant (female) requested that the driving test be stopped prematurely after use of l-mequitazine 5 mg combined with alcohol. Two participants did not start the driving test after l-mequitazine 10 mg combined with alcohol: one male participant due to nausea, and one female participant because the instructor had terminated her driving test after use of the drug alone. As a result, performance data from two participants are missing for l-mequitazine 10 mg combined with alcohol. For the six prematurely terminated tests, SDLP and SDSP scores were calculated from data recorded until termination.

### Blood alcohol concentrations

Alcohol challenges resulted in overall mean (±SD) blood alcohol concentrations (BACs) of 0.45 ± 0.09 mg/ml at the start of the second driving test, which declined to 0.30 ± 0.08 mg/ml at the conclusion of the driving test. Mean BACs measured in the placebo, l-mequitazine 2.5, 5.0 and 10 mg and mequitazine 10 mg conditions were 0.45, 0.48, 0.45, 0.47 and 0.43 mg/ml at the start of the second driving, and 0.31, 0.28, 0.29, 0.32 and 0.29 mg/ml at the end of the driving test, respectively. No significant differences were found in BACs between treatment conditions.

### Highway driving test

Analysis showed significant effects of Treatment (*F*_4.88_ = 4.30, *p* = 0.007) and Alcohol (*F*_1.22_ = 55.56, *p* < 0.001), on SDLP, but no significant interaction effect between Treatment × Alcohol (see Table 1). Mean (95 % CI) increases in SDLP after l-mequitazine 2.5, 5.0 and 10 mg, and mequitazine 10 mg alone, compared to placebo alone were 0.16 (−0.68; +1.01), 0.54 (−0.25; +1.33), 1.59 (+0.87; +2.32) and 0.33 (−0.37; +1.03) cm, respectively. Contrasts analysis showed that the effect of l-mequitazine 10 mg alone was highly significant compared to placebo (*p* < 0.001). Other simple contrasts of drug-placebo conditions were not significant.

**Table 1:** Mean (+SE) scores of the highway driving test, the AWLT and subjective measures per treatment, alone and with alcohol and summary of overall effects of treatment, alcohol and treatment × alcohol

	Highway driving		AWLT		Driving quality		
	SDLP	SDSP	Immediate	Delayed	Pre-test	Post-test	Alertness
No alcohol							
Plac	16.86 ± 0.56	2.15 ± 0.13	27.20 ± 1.24	8.62 ± 0.62	76.78 ± 5.34	72.13 ± 4.45	75.91 ± 3.68
L-meq 2.5	17.03 ± 0.49	2.23 ± 0.11	26.70 ± 1.84	8.24 ± 0.83	76.22 ± 4.73	65.17 ± 4.37	78.42 ± 2.90
L-meq 5.0	17.40 ± 0.69	2.19 ± 0.11	25.60 ± 1.51	8.19 ± 0.77	78.96 ± 4.37	64.39 ± 4.43	73.04 ± 3.11
L-meq 10	18.46 ± 0.59*	2.31 ± 0.12	24.40 ± 1.32	7.33 ± 0.52*	71.91 ± 4.62	62.74 ± 4.54	68.67 ± 3.55
Meq 10	17.19 ± 0.61	2.24 ± 0.13	24.30 ± 1.40	7.38 ± 0.66	75.30 ± 3.90	69.00 ± 3.48	75.39 ± 2.85
With alcohol							
Plac	19.40 ± 0.72	2.43 ± 0.14	20.60 ± 1.24	5.38 ± 0.71	62.00 ± 6.12	64.48 ± 4.29	63.39 ± 3.81
L-meq 2.5	19.68 ± 0.71	2.42 ± 0.14	21.55 ± 1.14	4.14 ± 0.69	51.83 ± 5.06	55.43 ± 5.24	58.71 ± 3.80
L-meq 5.0	19.63 ± 0.76	2.46 ± 0.14	22.05 ± 1.26	4.86 ± 0.64	59.43 ± 5.53	61.22 ± 3.49	59.39 ± 4.16
L-meq 10	20.81 ± 0.79^**⟡**^	2.52 ± 0.13	21.00 ± 1.15	3.90 ± 0.61	54.22 ± 5.36	59.17 ± 4.16	56.79 ± 4.07
Meq 10	20.57 ± 0.7^**⟡**^	2.56 ± 0.12	21.65 ± 1.36	4.43 ± 0.73	55.39 ± 4.36	55.04 ± 3.03	56.94 ± 3.50
N. total	23	23	20	21	23	23	21
ANOVA							
Treatment							
*df*	*4.88*	*4.88*	*4.76*	*4.80*	*4.88*	*4.88*	*4.80*
*F*	4.30	0.85	0.99	2.52	0.95	1.27	1.79
*p*	**0.007**	NS	NS	**0.048**	NS	NS	NS
Alcohol							
*df*	*1.22*	*1.22*	*1.19*	*1.20*	*1.22*	*1.22*	*1.20*
*F*	55.56	24.49	52.42	204.48	32.9	6.12	24.28
*p*	**<0.001**	**<0.001**	**<0.001**	**<0.001**	**<0.001**	**0.021**	**<0.001**
Treatment × alcohol							
*df*	*4.88*	*4.88*	*4.76*	*4.80*	*4.88*	*4.88*	*4.80*
*F*	1.88	0.46	2.12	0.47	0.63	1.01	2.40
*p*	NS	NS	NS	NS	NS	NS	NS

Significant treatment, alcohol or treatment × alcohol effects are indicated in bold (*p* < 0.05). Asterisks indicate: significant differences from placebo, *p < 0.05 (after sdHBC); significant differences from placebo with alcohol, ^⟡^ *p* < 0.05 (after sdHBC)

*Plac* placebo, *L-meq 2.5* l-mequitazine 2.5 mg, *L-meq 5.0* l-mequitazine 5.0 mg, *L-meq 10* l-mequitazine 10 mg, *Meq 10* mequitazine 10 mg, *NS* not significant, *df* degrees of freedom, *sdHBC* step-down Holm-Bonferroni correction

**Fig. 2:**
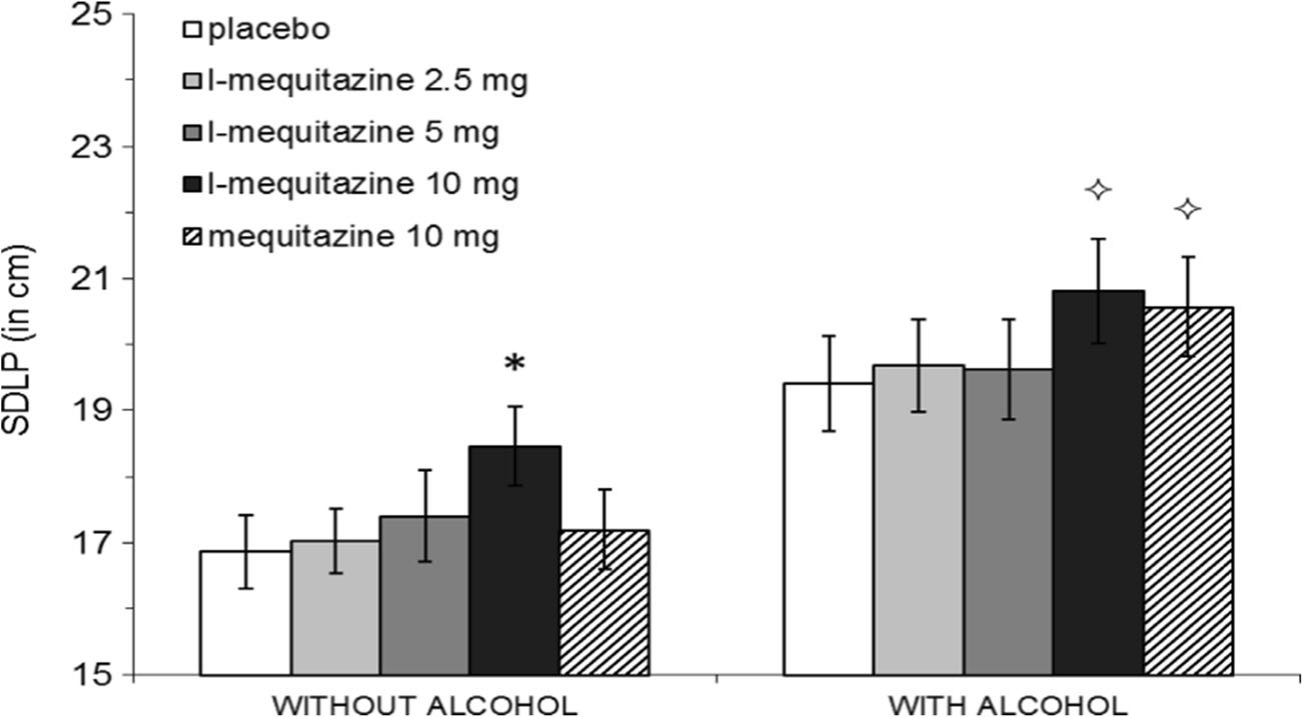
Mean (±SE) standard deviation of lateral position (SDLP) for each treatment, alone and with alcohol. *Asterisks above bars* indicate: significant differences from placebo, *p < 0.05; significant differences from placebo with alcohol, ^⟡^ *p* < 0.05

Alcohol significantly increased SDLP in all treatment conditions. Simple contrast analysis showed an overall mean (95 % CI) increase in SDLP of 2.54 cm (+1.99, +3.36) (see Fig. 2).

Mean (95 % CI) increase in SDLP after alcohol and l-mequitazine 2.5, 5.0 and 10 mg, and mequitazine 10 mg compared to alcohol alone were 0.29 (−0.90; +1.47), 0.22 (−0.67; +1.13), 1.41 (+0.54; +2.28) and 1.17 (+0.38; +1.96). Contrasts showed that the SDLP of both l-mequitazine 10 mg (*p* = 0.003) and mequitazine 10 mg (*p* = 0.006) in combination with alcohol was significantly larger then alcohol alone. Other drug-placebo contrasts were not significant.

For SDSP, a significant main effect of Alcohol was found (*F*_1.22_ = 24.49, *p* < 0.001). Alcohol significantly increased SDSP. Simple contrast analysis showed an overall mean (95 % CI) increase in SDSP of 0.26 cm (+0.15; +0.37).

### Memory

Mean (±SE) immediate and delayed recall score are shown in Table 1. Analysis showed a significant main effect of Alcohol on immediate recall scores (*F*_1.19_ = 52.42, *p* < 0.001), but no effects of Treatment. Alcohol significantly reduced the number of words in all treatment conditions. Simple contrast analysis showed an overall mean (95 % CI) decrease of −4.27 (−5.50; −3.04) words.

For delayed recall, repeated measures analysis showed a significant main effect of Treatment (*F*_4.,80_ = 2.52, *p* = 0.048), a significant main effect of Alcohol (*F*_1.20_ = 204.47, *p* < 0.001), but no significant interaction effect of Treatment × Alcohol. Mean (95 % CI) decrease in delayed recall scores after l-mequitazine 2.5, 5.0 and 10 mg, and mequitazine 10 mg alone compared to placebo were −0.38 (+1.24; −2.00), −0.43 (+0.91; −1.77), −1.29 (−0.33; −2.24) and −1.24 (+0.12; − 2.60) items, respectively. Contrast analysis showed that the effect of l-mequitazine 10 mg alone was highly significant (*p* = 0.011) compared to placebo. Other simple contrasts of drug-placebo conditions were not significant.

Alcohol significantly reduced the number of words recalled for delayed recall in all treatment conditions. Simple contrast analysis showed an overall mean (95 % CI) decrease of −3.41 (−3.91; −2.91) words. Mean (95 % CI) decrease in delayed recall score after alcohol and l-mequitazine 2.5, 5.0 and 10 mg, and mequitazine 10 mg compared to placebo with alcohol were −1.24 (+0.16; −2.63), −1.05 (+0.77; −1.82), −1.48 (−0.19; − 2.76) and −0.95 (+0.57; −2.48) items, respectively. Contrasts between treatment combined with alcohol and placebo in combination with alcohol showed no significant drug-placebo contrasts.

### Subjective evaluations

Analysis showed a significant effect of Alcohol for all subjective evaluations, but no effect of Treatment. After alcohol consumption, participants rated their expected driving ability and driving ability after completion of the driving test as significantly worse (*F*_1.22_ = 32.76, *p* < 0.001 and *F*_1.22_ = 6.12, *p* = 0.021, respectively). Alcohol significantly decreased alertness (*F*_1.20_ = 24.28, *p* < 0.001). An overview of the subjective evaluations is given in Table 1.

### Adverse events

No serious adverse events were recorded during the study. The most frequently reported adverse events were fatigue and headache. Fatigue was mainly observed after treatment with l-mequitazine 10 mg and mequitazine 10 mg. Headache was mainly observed in the placebo condition. A summary of the most commonly reported adverse events during all treatments is given in Table 2.

**Table 2:** Summary of total amount of adverse events after placebo, l-mequitazine 2.5 mg, l-mequitazine 5.0 mg, l-mequitazine 10 mg and mequitazine 10 mg as indicated by all participants who started treatment (*n* = 31). Only adverse events that are reported in more than 5 % of the cases are displayed

	Placebo	L-mequitazine 2.5 mg	L-mequitazine 5.0 mg	L-mequitazine 10 mg	Mequitazine 10 mg
Adverse event	*N* (%)	*N* (%)	*N* (%)	*N* (%)	*N* (%)
Fatigue	3 (9.7)	4 (12.9)	3 (9.7)	6 (19.4)	6 (19.4)
Headache	6 (19.4)	3 (9.7)	1 (3.2)	4 (12.9)	5 (16.1)
Nausea	3 (9.7)	2 (6.5)	–	3 (9.7)	–
Somnolence	1 (3.2)	3 (9.7)	2 (6.5)	1 (3.2)	3 (9.7)
Vomiting	1 (3.2)	2 (6.5)	–	–	1 (3.2)

## Discussion

The current study was conducted to assess the effects of single doses of l-mequitazine (2.5, 5.0 and 10 mg) and mequitazine (10 mg), alone and in combination with alcohol, on actual highway driving performance. Results indicate that l-mequitazine, 2.5 and 5.0 mg, had no effect on highway driving performance. L-mequitazine 10 mg impaired highway driving performance, as indicated by the significant 1.6-cm increase of SDLP. Mequitazine 10 mg showed no effect on highway driving performance. With regard to memory performance, mequitazine 10 mg and l-mequitazine 2.5 and 5.0 mg showed no effect. L-mequitazine 10 mg only impaired delayed recall. The difference in impairing effects on driving performance and memory of equal doses (10 mg) of l-mequitazine and mequitazine could be explained by l-mequitazine’s higher affinity for histamine H_1_ receptors (Gauthier et al., 2014; Renault and Le, 1983). Given that l-mequitazine’s binding affinity for muscarine receptors is low as compared to mequitazine, the effects cannot easily be explained by anticholinergic activity of these drugs (Clerc et al., 2012; Heusler et al., 2015).

The clinical relevance of drug-induced performance changes in the highway driving test is often determined by referring to the relationship between blood alcohol concentration (BAC) and SDLP (Louwerens et al., 1987), which showed that an average SDLP increase of 2.4 cm is comparable to driving under the influence of a BAC of 0.5 mg/ml. Acute effects of l-mequitazine 10 mg caused a significant increase in SDLP of 1.6 cm compared to placebo. Although this increase is significant, this effect can be considered mild, given that the SDLP increase between the alcohol-placebo and placebo treatment is 2.5 cm in the current study. The combination of any treatment with alcohol led to significant performance impairment. On average across all treatments, alcohol increased SDLP by 2.63 cm, and decreased immediate and delayed recall by 4.27 words and 3.41 words, respectively. The effects of alcohol and drugs did not interact significantly. This indicates that, although the effects of alcohol added to the effects of the drugs, the combined use did not result in disproportionally increased impairment (i.e. potentiation of effects). This lack of potentiation is in line with previous studies assessing the combined effect of alcohol and antihistamines (Vermeeren and O’Hanlon, 1998; Vermeeren et al., 2002a).

Memory impairment was observed after administration of l-mequitazine 10 mg alone on delayed recall. This could be due to indirect effects of H_1_ receptor blockade (Van Ruitenbeek et al., 2010), such as impaired concentration and fatigue (Lieberman III, 2004; Penttilä et al., 2005), since headache and fatigue were reported as adverse events in the l-mequitazine condition. Lower dosages of l-mequitazine did not impair immediate or delayed recall, alone or with alcohol intake.

Participants were not aware of the driving impairment caused by antihistamines, which is in line with previous studies (Theunissen et al., 2006; Vermeeren et al., 2002a). Participants were, however, aware of the impairing effects of alcohol on driving performance, which is also in line with a previous study (Vermeeren et al., 2002b). For l-mequitazine 10 mg, participants were unable to predict their impairment before driving and were unaware of the impairing effects after test completion. Given that the effect of l-mequitazine 10 mg was not clinically relevant, it can be assumed that the effect was not large enough to be noticed by the participants.

The increase of SDLP (+0.33 cm) after mequitazine 10 mg alone was smaller than reported in previous placebo-controlled studies, which showed a mean SDLP increase of 2.5 cm (Theunissen et al., 2006) and 1.0 cm (Theunissen et al., 2004). The smaller effect found in the current study could be due to the exclusion of cytochrome P450 CYP 2D6 slow metabolizers and due to the start of the first driving test, i.e. 1 to 2 h after the *t*_max_ of mequitazine. However, given the low prevalence of slow metabolizers (10 % in the Caucasian population) (Tamminga et al., 2001) and the long half-life (18 h) of mequitazine, findings from this study might implicate that the impairing effect of mequitazine 10 mg is, on average, milder than previously reported.

In conclusion, l-mequitazine 10 mg produced mild driving impairment, whereas l-mequitazine 2.5 and 5.0 mg show no effects on driving. It should be noted that the absence of effects only reflects the average individual and that individual differences should be taken into account. Alcohol (BAC of 0.5 mg/ml) led to significant driving impairment, but did not potentiate the effects of l-mequitazine or mequitazine.
